# Phase II Trials of Iniparib (BSI-201) in Combination with Gemcitabine and Carboplatin in Patients with Recurrent Ovarian Cancer

**DOI:** 10.1093/oncolo/oyac275

**Published:** 2023-01-30

**Authors:** Richard T Penson, Allison J Ambrosio, Christin A Whalen, Carolyn N Krasner, Panagiotis A Konstantinopoulos, Charles Bradley, Ursula A Matulonis, Michael J Birrer

**Affiliations:** Department of Medicine, Massachusetts General Hospital, Boston, MA, USA; Department of Medicine, Massachusetts General Hospital, Boston, MA, USA; Department of Hematology Oncology, Dana-Farber Cancer Institute, Boston, MA, USA; Department of Medicine, Massachusetts General Hospital, Boston, MA, USA; Department of Hematology Oncology, Beth Israel Deaconess Medical Center, Boston, MA, USA; Department of Hematology Oncology, Dana-Farber Cancer Institute, Boston, MA, USA; BiPar Sciences, Inc., South San Francisco, CA, USA; Annexon, Inc., Brisbane, CA, USA; Department of Hematology Oncology, Dana-Farber Cancer Institute, Boston, MA, USA; Department of Medicine, Massachusetts General Hospital, Boston, MA, USA; P. Rockefeller Cancer Institute, Little Rock, AR, USA

## Abstract

**Background:**

Iniparib (BSI-201), a novel anticancer agent thought to have poly(ADP-ribose) polymerase (PARP) inhibitory activity and synergy with both gemcitabine and carboplatin (GC) was evaluated in 2 cohorts with GC.

**Methods:**

Parallel multicenter, single-arm, phase II studies using a Simon two-stage design. Eligible patients had a histological diagnosis of epithelial ovarian carcinoma, fallopian tube cancer, or primary peritoneal carcinoma and demonstration of platinum-sensitive (≥6 months [mo]) or -resistant disease (relapse 2-6 mo post-platinum). Carboplatin (AUC 4 IV day 1), gemcitabine (1000 mg/m^2^ IV days 1 and 8), and iniparib (5.6 mg/kg IV days 1, 4, 8, and 11) were given on a 21-day cycle.

**Results:**

The overall response rate (ORR RECIST 1.0) in platinum sensitive disease was 66% (95% CI, 49-80) with a higher response rate in the 15 pts with germline *BRCA* mutations (g*BRCA*^mut^) (73%). Median PFS was 9.9 (95% CI, 8.2-11.3) months. In the platinum resistant population the ORR was 26% (95% CI, 14-42), however in the 11 pts for whom *BRCA* mutation was present, the best overall response was PR in 5 (46%). Median PFS was 6.8 months (range, 5.7-7.7 months). Notably, among the 17 CA-125-response-evaluable patients who did not achieve tumor response, 7 (41.2%) patients had a CA125 response, and 93% has clinical benefit (CR + PR + SD). The GCI combination was generally well tolerated despite a high incidence of thrombocytopenia and neutropenia, with no new toxicities.

**Conclusions:**

Given the subsequent lack of efficacy demonstrated for iniparib in breast cancer, these are studies of GC and demonstrate a higher than traditionally appreciated activity in patients with platinum-sensitive and -resistant recurrent ovarian cancer, especially in patients that harbor a *BRCA* mutation, resetting the benchmark for efficacy in phase II trials. (ClinicalTrials.gov Identifiers: NCT01033292 & NCT01033123).

## Teaser

This article evaluates the potential efficacy of iniparib in combination with gemcitabine and carboplatin in platinum-sensitive ovarian cancer.

**Implications for Practice**

Controlled trials are essential to define standards of care.

## Background

Ovarian cancer is a devastating disease with the highest mortality rate of all gynecologic tumors, with an estimated 22 280 new diagnoses and 14 240 deaths annually in the United States, and is the 8th most common cancer in women worldwide.^1^ There is no effective screening strategy, and early symptoms are subtle, so most cases present with advanced disease and a poor prognosis.^2^

Approximately one-third of recurrences occur within 6 months of last platinum and are arbitrarily defined as “platinum resistant,” while the majority occur later and are potentially platinum sensitive. The Gynecologic Cancer Inter-Group (GCIG) has helpfully defined 4 categories: platinum refractory (radiologically confirmed progressive disease (PD) on platinum or within one month), resistant (PD in >1-<6 months), partially sensitive (6-12 months), and sensitive (>12 months).^3^

Pfisterer et al. conducted the randomized controlled trial (RCT) through the AGO (Arbeitsgemeinschaft Gynaekologische Onkologie) which demonstrated a progression free survival (PFS) advantage for gemcitabine plus carboplatin over single agent carboplatin in patients with platinum-sensitive recurrent ovarian cancer,^4^ that led to FDA approval of gemcitabine for ovarian cancer in 2006. In that study, adding gemcitabine improved the ORR from 31% to 47% (*P* = .0016).

Iniparib (4-iodo-3-nitrobenzamide) is a novel, highly lipophilic investigational anticancer agent that distributes rapidly and widely into tissues. It is metabolized via a nitro-reduction pathway to a potent nitroso metabolite that binds covalently and irreversibly to PARP1.^5^ Preclinical studies demonstrated that Iniparib was mutagenic, and neurobehavioral effects were predicted to be the dose-limiting toxicity. At the time of these studies, iniparib had been demonstrated to induce cell-cycle arrest in the G2/M phase, and potentiation of cell-cycle arrest induced by DNA damaging agents, including platinum and gemcitabine. However, although it induced γ-H2AX foci it did so at drug concentrations that did not demonstrate PARP inhibition, and the physiologic targets of iniparib and its metabolites remain unclear.^6^ Preliminary clinical data did not identify any dose-limiting toxicity escalating to 8.0 mg/kg in phase I, and suggested improved efficacy outcomes with gemcitabine and carboplatin in a randomized phase II study in patients metastatic triple-negative breast cancer that fueled excitement about synergistic benefit with the combination of iniparib and GC as the first putative PARP inhibitor in phase III trial.^7^

PARP1 is a critical enzyme in DNA repair and may play a role in resistance to chemotherapy. Inhibiting PARP exploits the synthetic lethality of impaired homologous recombination set up by *BRCA* mutations, or the BRCA-ness present in many high-grade serous ovarian cancers.^8^

These 2 studies were designed to evaluate the potential efficacy of this combination in platinum sensitive disease and whether the addition of iniparib and gemcitabine could overcome platinum resistance.

## Methods

The studies underwent formal IRB review and were registered at clinicaltrials.gov (Sensitive: NCT01033123; Resistant: NCT01033292). Each study was designed as a multi-center, single-arm phase 2 using a Simon 2-stage design. In both studies, the primary endpoint was objective response rate (ORR), evaluated in all patients who received at least 1 dose of study drug and had 2 post-baseline assessments or progression/death within 60 days of last assessment. Secondary endpoints were progression free survival (PFS) and safety using NCI-CTCAE v3.0. A CA125 response was defined as 50% fall in CA125 initially >2× ULN and maintained for at least 28 days. A prospectively planned exploratory analysis of the relation between *BRCA* status and response was undertaken.

### Treatment

The regimen (GCI) consisted of gemcitabine given without fixed-dose rate adjustment over 30 min at 1000 mg/m^2^ days 1 and 8, followed by carboplatin AUC 4 on day 1, with iniparib 5.6 mg/kg on days 1, 4, 8, and 11 every 3 weeks (Q21) as a 60-min IV infusion. Treatment with GCI was planned for at least 6 cycles in the absence of PD or unacceptable toxicity, and patients could be continued for an additional 4 cycles at physician’s discretion. Iniparib could be continued beyond 10 cycles as maintenance until PD. Participants had to achieve an ANC ≥1000/mm^3^ and/or platelet count ≥100 000/mm^3^ to receive chemotherapy. Dose reductions we designed keeping the carboplatin dose at AUC 4, and reducing gemcitabine from 1000 mg/m^2^ to 800 mg/m^2^ for grade 3 toxicity, and D8 gemcitabine was discontinued for a second episode of any of the following: febrile neutropenia, ANC ≤500/mm^3^ for >5 days in a cycle, ANC ≤100/mm^3^ for >3 days in a cycle, platelets <50 k, bleeding associated with thrombocytopenia, and day 1 delayed for >2 weeks. Treatment could be held for up to 3 weeks for any reason. There were no iniparib dose reductions planned, and participants with grades 3 or 4 toxicity that required a dose to be held could continue to receive iniparib.

### Patient Eligibility

Key eligibility criteria included having epithelial ovarian cancer, fallopian tube cancer, or primary peritoneal carcinoma, measurable disease per RECIST 1.1 criteria, and ECOG performance status 0-2.

Platinum-sensitive disease was defined as radiological relapse >6 months after the last dose of platinum or platinum-based chemotherapy and required that participants had received no prior cytotoxic chemotherapy in the recurrent setting. Platinum-resistant disease was defined as radiologically confirmed relapse within 2-6 months of platinum-based therapy, and patients could have at least 1 but not more than 2 prior therapies. Patients with or without *BRCA* mutations were eligible. Adequate organ function defined as ANC ≥ 1500/mm^3^, platelets ≥ 100 000/mm^3^, creatinine clearance >50 mL/min (estimated using the Cockcroft-Gault formula), ALT and AST <2.5× upper limit of normal (ULN; or <5× ULN in case of liver metastases); total bilirubin <1.5 mg/dL. Patients also had to be ≥18 years of age, and the study stipulated typical requirements for contraception, comorbidities, other malignancies, and prior biologics counted as lines of treatment if used for >6 months, excluding hormones. Prior treatment with PARP inhibitors was an exclusion criterion.

### Statistics

For the platinum sensitive study, we used the AGO registration study as a historical control with an ORR of 47% and the trial sample size was designed to detect an improvement in ORR from 40% to 60%.^4^ More than 8 responses were required in the initial 17 patient cohort to proceed to stage 2 of the platinum sensitive study, with a further 24 patient enrolled (*n* total = 41). The platinum resistant study required 23 (stage 1) and 25 (stage 2) participants, respectively, and was powered to detect an improvement in ORR from 15% to 30% based on an accepted historical control ORR of ~15% in this platinum-resistant patient population.^9^ Best overall response (CR, PR, stable disease (SD), PD, not evaluable (NE)) and ORR were summarized using descriptive statistics, and 95% CI was calculated for ORR. A waterfall plot of maximum percent reduction from baseline in tumor burden of target lesions was produced. PFS was analyzed using the Kaplan–Meier method and summarized with median and 95% CIs of the median.

## Results

### Accrual and Treatment

The sensitive study enrolled all 41 patients between December 9, 2009 and February 12, 2012. The resistant study enrolled 46 of the planned 48 patients between December 11, 2009 and December 16, 2012, and 1 patient though enrolled, did not receive any study medication. Sixteen patients (39%) with sensitive disease continued iniparib as maintenance therapy. This number was only 8 (18%) for resistant patients. Demographics are shown in Table 1. For both studies the median number of chemotherapy cycles administered was 6 (1-11 for sensitive and 1-10 for resistant). The median number of iniparib cycles administered was 9 (3 to 22) for sensitive and 8 (1 to 16) for resistant.

**Table 1.** Demographics.

	Platinum sensitive *n* = 41	Platinum resistant *n* = 46
Age, years, median (range)	59 (35-82)	61 (37-85)
ECOG PS (%) 0/1/2	58%/42%/0	27%/70%/3%
Platinum-free interval, months, median (range)	12.6 (7-74)	4.6 (2-6)
6-11 month; 12-24 month; ≥24 months	46%; 37%; 17%	
Tumor histology, %		
Ovarian/peritoneal/FTube/UKN	73%/14%/7%/6%	76%/4%/20%/0%
Tumor grade, 1/2/3	0%/9%/91%	2%/7%/91%
Histology, *n* (%)		
Serous	35 (85%)	29 (63%)
Endometrioid	1 (2.4%)	3 (7%)
Clear cell	1 (2.4%)	4 (9%)
Other^*^	4 (10%)	10 (22%)

^*^In the platinum resistant group: 7 mixed endometrioid and serous and 3 carcinosarcomas (MMMT).

### Efficacy

**Figure 1.**
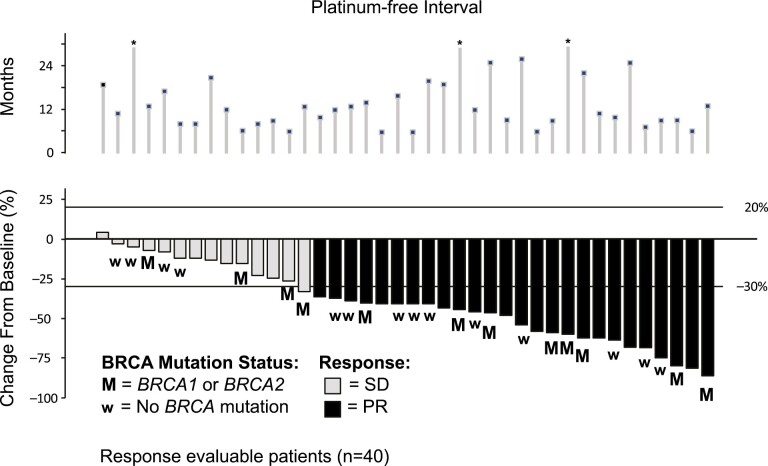
Objective response in platinum sensitive GCI patients.

**Figure 2.**
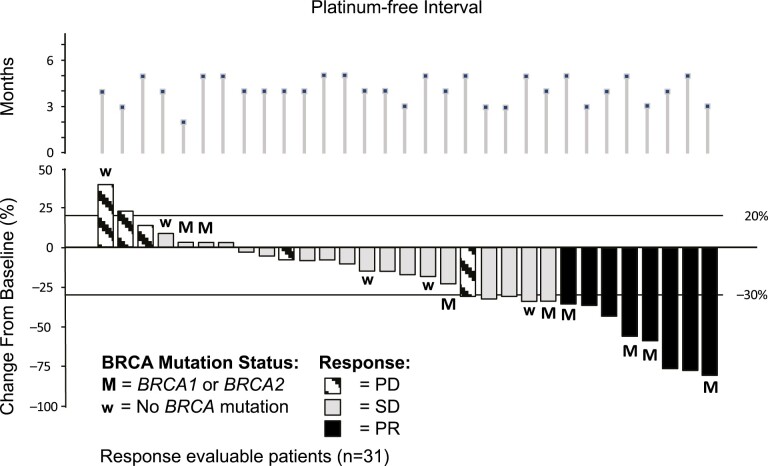
Objective response in platinum resistant GCI patients.

**Figure 3.**
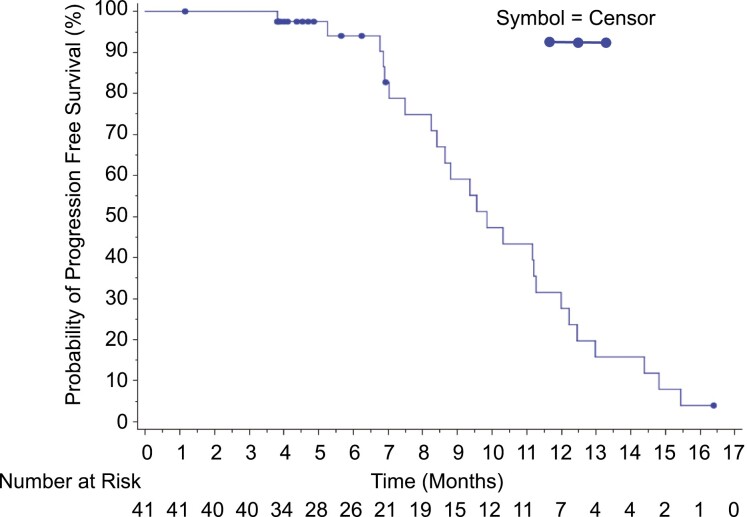
PFS in platinum sensitive GCI patients.

**Figure 4.**
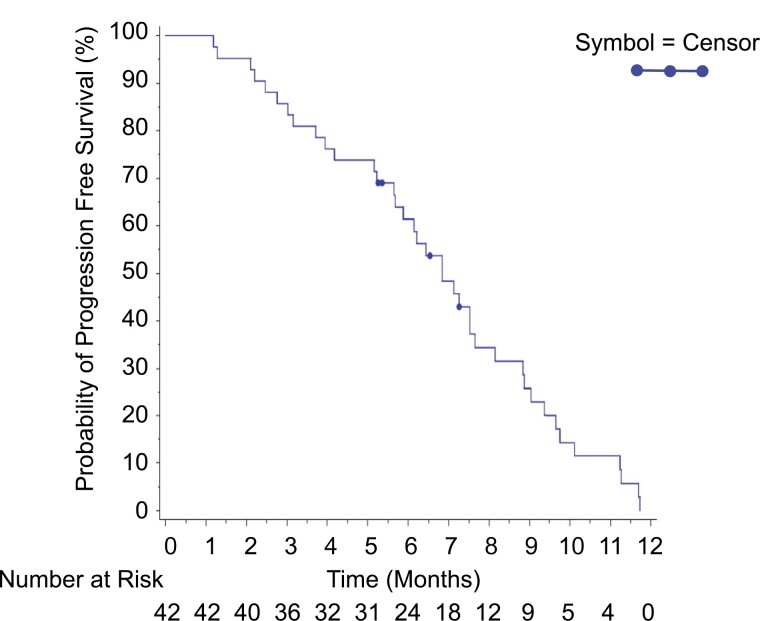
PFS in platinum resistant GCI patients.

Best overall response is shown in Table 2. In the platinum sensitive patient population GCI produced a confirmed overall response rate (ORR; RECIST 1.0) in platinum sensitive disease was 66% (95% CI, 49.4-79.9) with responses seen in both *BRCA* mutant and wild-type patients (Fig. 1). Median PFS was 9.9 (95% CI, 8.2-11.3) months. Only 18 patients were considered evaluable for CA-125 response (ie, patients who had a pretreatment sample that was at least twice the ULN taken within 2 weeks prior to starting treatment), of whom 14 (77.8%) had a CA-125 response (Figs. 2-4).

**Table 2.** Response data.

Best response	Platinum sensitive *n* (%)		Platinum resistant *n* (%)	
	Response evaluable (*n* = 41)	g*BRCA*^mut^ (*n* = 15) (absent in 15 UNK in 11)	Response *n* = 45	g*BRCA*^mut^*n* = 11 (24%) (absent in 14 UNK in 20)
CR	0	0	0	0
PR	27 (66%)	11 (73.3%)	11 (26%)	5 (46%)
SD	13 (31.7)	4 (26.7%)	28 (67%)	NA
PD	1 (2.4%)	0	3 (7%)	NA
Inevaluable			6 (13%)*	0
ORR (CR + PR)	66%	73%	26%	45%
Clinical benefit rate (CBR) (CR + PR + SD)			93%	

Inevaluable patients: *5 for withdrawal of consent and 1 with SAE.

In the platinum resistant population the ORR was 26.2% (95% CI, 13.9-42.0). Among the 14 patients for whom *BRCA* mutation was reported as absent, the best overall response was PR in 4 (28.6%) patients. Among the 11 patients for whom *BRCA* mutation was reported as present, the best overall response was PR in 5 (45.5%) patients. Progression-free survival was available for 38 (90.5%) of the patients with a PFS event (ie, had clinical or radiological progression or died) and median PFS was 6.8 months (range, 5.7-7.7 months). Notably, among the 17 CA-125-response-evaluable patients who did not achieve tumor response, 7 (41.2%) patients had a CA125 response.

### Toxicity

Safety profiles consistent with those observed in prior clinical studies with GC. There were no deaths on study or within 60 days of the last dose of study drug. In the sensitive study 12 patients (29%) experienced a serious treatment fmergent adverse event (SAEs), and the most common were neutropenia and thrombocytopenia, 5 (12%) SAEs were considered related to study drug (thrombocytopenia (2 pts), neutropenia, urinary tract infection, and pulmonary embolism). In the resistant study 22 patients (49%) experienced a SAE and in 9 patients (26%) the SAEs were considered related to study drug (thrombocytopenia (2 pts), anemia, nausea, vomiting, small intestinal obstruction, fatigue, dehydration, and hypokalemia), and in 4 (9%) this lead to discontinuation of treatment. Toxicity is shown in Table 3.

**Table 3.** Toxicity.

Toxicity	Platinum sensitive		Platinum resistant	
	All grades %	Grade ¾ %	All grades %	Grade ¾ %
Fatigue	90	0	89	6
Neutropenia	81	59	80	73
Nausea	76	2	84	14
Constipation	66	0	64	0
Thrombocytopenia	56	42	62	38
Anemia	42	5	69	14
Vomiting	29	2	60	6
Drug hypersensitivity	29	0	20	3
Neuropathy peripheral	29	2	17	0
Pyrexia	29	0	20	3
Diarrhea	27	7	29	3
Hypomagnesaemia	24	0	20	0
Back pain	20	0	20	0
Headache	20	0	13	0
Rash	20	0	13	0
Alopecia	17	NA	9	NA
Dysguesia	15	0	0	0
Anxiety	15	0	24	0
Abdominal pain	15	2	24	9
Infection	15	2		

Toxicity, TEAE (treatment emergent adverse event) occurring in 15%.

Dose reductions or delays due to an AE were common and almost all due to hematologic toxicity (89% for platinum sensitive and 91% for platinum resistant). Dose reductions and delays 81% and 32%, respectively, for platinum sensitive; 85% and 27% for platinum resistant patients. The majority of patients in the platinum sensitive group received >6 cycles: 22 (54%) of the patients received 7-12 cycles, two (5%) 10-18 cycles, and one >18 cycles of therapy. Thirty of the platinum sensitive patients (73%) received G-CSF whereas this was only required in 27% of the platinum resistant patients.

## Discussion

These phase II clinical trials investigated a promising novel agent, thought to be a PARP inhibitor with potential synergy with gemcitabine and carboplatin in 2 cohorts of patients with recurrent ovarian cancer. Subsequent studies have clarified the lack of even additive benefit, and the mechanism of in vitro cytotoxicity observed with iniparib remains obscure.^10^ The clinical development of new agents remains a pressing need and the clinical trials structure is the optimal way to ensure the fair and most expeditious way to test promising new drugs. Our experience has been a sobering reminder of the need for critical review of all the data, while retaining a healthy skepticism, given the large number of hypotheses and novel compounds that prove to be disappointing.

Sanofi purchased iniparib from BiPar Sciences Inc. in 2009, 2 years before we presented these data at the American Society of Clinical Oncology (ASCO) annual meeting, at a cost of up to $500 million if iniparib met its development targets.^11^ The phase III data was presented in 2011 (breast) and 2013 (lung) ASCO conferences, and at the latter, Sanofi disclosed that iniparib would no longer be developed, and a phase I trial exploring higher doses was abandoned.^12^

Gemcitabine and carboplatin remains a standard combination with proven efficacy in platinum sensitive recurrent disease,^4^ and serves as a particularly good platform for the evaluation of novel biologics.^13^ We likely met the proscribed end point for platinum sensitive disease (>60% ORR) because of the greater number of patients recruited with *BRCA* mutations. Furthermore, the reasonably good response rate in platinum resistant disease speaks to the potential for this combination to be evaluated with new agents that may overcome resistance given the relative lack of non-hematologic toxicity that impair quality of life (HRQoL), such as hair loss and neuropathy.

It is noteworthy that almost one quarter of the platinum resistant group carried a g*BRCA*^mut^, and that this group had a much higher response to platinum (46%), despite using the typical criteria, and *BRCA* status clearly influences response to platinum. There has been concern that platinum resistant patients may not respond to PARP inhibitors,^14^ but combination regimens may have a better than expected response compared to historical controls, and BRCA status (g*BRCA*^mut^ or s*BRCA*^mut^ (somatic or “tissue”)), or so-called BRCA-ness through other genetic or epigenetic disruption may be key determinants of response.

In summary, GCI produced a confirmed ORR of 66% in the platinum sensitive patient population and 26% in the platinum resistant population. The GCI combination was generally well tolerated despite high incidence of thrombocytopenia and neutropenia, with no new toxicities, and safety profiles consistent with those observed in prior clinical studies with GC. Higher than expected response rates should be anticipated when there are selective pressures, such as the promise of benefit from a new investigational agent, heralded as a first in class breakthrough, that disproportionately draws in more patients with BRCA mutated tumors. For example, the SOLO3 trial which evaluated the PARP inhibitor, olaparib as a single agent against non-platinum based single agent chemotherapy in patients with *BRCA* mutated platinum sensitive recurrent ovarian cancer, the subgroup who had received only 2 prior lines of treatment had an ORR of 85%.^15^ Cognizant of the selection bias of phase II trials, phase III trials remain the true benchmark for defining standard of care.

## Data Availability

The data underlying this article will be shared on reasonable request to the corresponding author.
